# Diagnostic accuracy and clinical impact of filmarray multiplex PCR system in bloodstream infections: A comparative study with conventional methods in a tertiary health care setting

**DOI:** 10.1097/MD.0000000000043263

**Published:** 2025-07-18

**Authors:** İsmail Selçuk Aygar, Tuğrul Hoşbul

**Affiliations:** a Medical Microbiology Laboratory, Gulhane Training and Research Hospital, Health Sciences University, Ankara, Keçiören, Türkiye; b Department of Medical Microbiology, Health Sciences University Gulhane Faculty of Medicine, Ankara, Türkiye.

**Keywords:** antimicrobial resistance, bloodstream infection, rapid molecular diagnostics

## Abstract

The prompt identification of the causative microorganism responsible for bloodstream infections (BSIs) is paramount, as is the determination of its resistance profile. The aim of this study was to evaluate the diagnostic performance of the BioFire FilmArray Blood Culture Identification 2 (BCID2) panel for BSIs compared to conventional methods (CTMs) and its impact on antimicrobial treatment decisions. The study included 93 positive blood cultures from 85 patients with suspected BSI between January and August 2024. The BCID2 system demonstrated an accuracy of 90.32% in identifying positive samples in culture. The analytical sensitivity was calculated to be 83.72%, while the clinical sensitivity was determined to be 77.41% at the sample level. The highest concordance at the isolate level was observed for *Klebsiella pneumoniae*, *Serratia marcescens*, and *Acinetobacter baumannii*, with a 100% concordance rate. The compatibility of the method with conventional techniques was found in 85.13% of monomicrobial samples and 47.36% of polymicrobial samples. The mean time to result was determined as 1 day and 4 hours for BCID2 and 2 days and 4 hours for CTMs to identification; the difference was found to be significant. With regard to the detection of antimicrobial resistance genes, a 73.46% concordance was observed between BCID2 and conventional phenotypic methods at the sample level. While the presence of resistance was detected phenotypically in *A. baumannii* isolates, this could not be demonstrated genotypically with BCID2. In treatment simulations for antimicrobial stewardship, 91.5% of correct treatment arrangements were achieved according to the Norwegian guideline. Furthermore, an examination of the impact of gram stain pattern on diagnostic agreement revealed 90% agreement in anaerobic bottles, 77.77% in pediatric bottles, and 73.43% in aerobic bottles. However, these differences were not found to be statistically significant. Conversely, a significant difference in agreement rates was observed according to gram stain pattern, with 94.1% agreement observed in gram-positive cocci and 79.1% in gram-negative bacilli, yielding a statistically significant difference. BCID2 has the potential to improve clinical decision-making processes in the management of BSIs with its rapid diagnosis time, high specificity, and resistance detection capacity; however, due to its current limitations, it is currently best used as a supportive tool to CTMs.

## 1. Introduction

Bloodstream infections (BSIs) are serious and life-threatening conditions associated with high morbidity, mortality, and economic burden. Timely initiation of appropriate antimicrobial therapy is essential for optimal clinical outcomes in the treatment of patients. However, patients are often empirically treated with a combination of antimicrobial agents until the causative pathogen is accurately identified, and this approach may not always be appropriate.^[1,2]^

National and international guidelines recommend the initiation of intravenous antimicrobial therapy within the first hour after diagnosis of sepsis to improve patient outcomes and reduce the risk of mortality.^[3]^ Rapid identification of the pathogen is necessary for the transition from empirical treatment to targeted antimicrobial therapy; however, conventional blood culture (BC) methods cause delays in diagnosis, necessitating the need for faster diagnostic tools in BSI.^[4]^ Urgent identification of the pathogen and determination of its antimicrobial susceptibility profile are of great importance. Rapid BC identification panels developed to overcome this problem are based on polymerase chain reaction (PCR) and offer a promising option by allowing detection of pathogens and resistance genes within 1 to 2 hours.^[5]^ Conventional microbiological techniques can take up to 72 hours to identify the causative microorganism and determine its susceptibility.

Recent advances in multiplex PCR-based diagnostics have significantly improved the rapid identification of microorganisms in BCs, allowing timely initiation of targeted antimicrobial therapy and optimizing the management of BSIs.^[6]^ In addition, they are distinguished by their ability to rapidly and accurately detect antimicrobial resistance (AMR)-associated genes, thereby increasing diagnostic sensitivity (Sn) and helping to guide appropriate treatment.^[7,8]^ This approach accelerates the initiation of appropriate antibiotic therapy, strengthens antimicrobial surveillance practices, and reduces the risk of resistance development by reducing the overuse of broad-spectrum antibiotics.^[5,8–10]^ However, studies of the clinical utility of these diagnostic tools and their impact on hospital outcomes and mortality have produced conflicting results.^[8,9,11,12]^

BioFire FilmArray Blood Culture Identification 2 (BCID2) (BioFire Diagnostics, bioMérieux, Salt Lake City, Utah), 1 bacterial order (*Enterobacterales*), 4 bacterial genera (*Proteus spp.*, *Salmonella spp.*, *Staphylococcus spp*, *Streptococcus spp*.), 21 bacterial species (*Acinetobacter calcoaceticus-baumannii complex*, *Bacteroides fragilis*, *Enterobacter cloacae complex*, *Enterococcus faecalis*, *Enterococcus faecium*, *Escherichia coli*, *Klebsiella aerogenes*, *Klebsiella oxytoca*, *Klebsiella pneumoniae group*, *Listeria monocytogenes*, *Serratia marcescens*, *Haemophilus influenzae*, *Neisseria meningitidis*, *Pseudomonas aeruginosa*, *Staphylococcus aureus*, *Staphylococcus epidermidis*, *Staphylococcus lugdunensis*, *Stenotrophomonas maltophilia*, *Streptococcus agalactiae*, *Streptococcus pneumoniae and Streptococcus pyogenes*), 1 fungal genus (*Cryptococcus* (*C. neoformans*/*C. gattii*) and 6 fungal species (*Candida albicans*, *Candida auris*, *Candida glabrata*, *Candida krusei*, *Candida parapsilosis*, *Candida tropicalis*). It also detects many AMR genes, including beta-lactamases (*bla*_CTX-M_ [ESBL]), carbapenemases (*bla*_IMP_, *bla*_KPC_, *bla*_OXA48-like_, *bla*_NDM_, *bla*_VIM_), methicillin resistance (*mecA*/*C* (MRSA), *mecA*/*C*, *mrej* [for MSSE, *Staphylococcus epidermidis*]), vancomycin resistance (*vanA*/*B* - VRE), and colistin resistance (*mcr-1*).

In cases of suspected sepsis, empiric treatment is usually initiated with broad-spectrum antibiotics selected based on regional endemic microorganisms, regional resistance patterns, and the potential source of infection identified by clinical findings. The choice of antibiotics varies between countries. In countries such as the UK (Stanford University) and Norway, various guidelines are available to guide the initiation of empiric treatment, de-escalation, or optimization of resistance genes or specific pathogens.^[13,14]^

The aim of this study was to evaluate the performance of the BioFire FilmArray BCID2 (BioFire Diagnostics) panel in comparison with conventional diagnostic methods in terms of species identification and resistance detection, and to investigate the impact of the BCID2 panel on clinical decision making. Some previous studies have evaluated the effectiveness of the BCID2 panel in the rapid identification of bloodstream pathogens and AMR markers.^[7,15,16]^ In addition, our study aimed to simulate the impact of BCID2 results on antimicrobial surveillance practices in patients and their possible implications for early initiation of optimal antimicrobial therapy by referring to 2 different guidelines.

## 2. Methods

This retrospective observational study was conducted between January and August 2024 at our tertiary care hospital. It included BC samples from hospitalized patients of all age groups with clinical suspicion of sepsis. Data were retrieved from the hospital information management system. Each sample was anonymized using a unique identifier.

BC was accepted as the gold standard method. Paired aerobic and anaerobic bottles were incubated in the BacT/ALERT 3D system (bioMérieux, France) for up to 5 days or until flagged positive. All positive samples were gram-stained and identified using MALDI-TOF MS (Bruker Daltonics, Germany). Antimicrobial susceptibility testing was conducted using conventional methods (CTMs) and the automated VITEK 2 system (bioMérieux, France), interpreted according to EUCAST guidelines.^[17]^

For molecular testing, the BioFire® FilmArray® BCID2 panel (bioMérieux, Utah, USA) was used to detect 26 bacterial species, 7 fungal species, and 10 AMR genes directly from positive BC bottles. Results were automatically recorded in the hospital information system. For patients with >1 positive culture within a 2-week period yielding the same organism, only the first sample was included. In total, 93 samples from 86 patients were analyzed.

Samples without antimicrobial susceptibility testing results, polymicrobial cultures with identical susceptibility profiles, and BCID2-negative samples were excluded from the AMR gene performance evaluation. Diagnostic performance was assessed at both the sample and isolate levels. At the sample level, overall agreement with CTMs was determined. At the isolate level, each identified pathogen was compared individually. Comparisons restricted to BCID2 panel organisms were defined as “analytical,” while those including non-panel organisms were labeled “clinical.”

Diagnostic parameters, including Sn, specificity (Sp), positive predictive value, negative predictive value, and concordance rate, were calculated using the formulas shown in Table 1. False negatives (FNs) were defined as targets detected by culture but missed by BCID2; false positives (FPs) as BCID2 detections not confirmed by CTMs.

**Table 1:** Formulas applied for comparison.

Sn (%)	(TP)/(TP + FN) × 100
Sp (%)	(TN)/(TN + FP) × 100
PPV (%)	(TP)/(TP + FP) × 100
NPV (%)	(TN)/(TN + FN) × 100
Concordance (%)	(TP + TN)/(TS) × 100

FN = false negative, FP = false positive, NPV = negative predictive value, PPV = positive predictive value, Sn = sensitivity, Sp = specificity, TN = true negative, TP = true positive.

The turnaround times for BCID2 and CTMs were also compared. “BCID2 result time” was defined as the time to automated reporting of the molecular result, while “culture result time” referred to the time to species identification via conventional workflow.

Finally, empirical treatment simulations were performed using national guidelines from Norway^[13]^ and the UK (Stanford University),^[14]^ based on organism identification and resistance gene detection from BCID2 results.

The study received approval from the Health Sciences University Gülhane Scientific Research Ethics Committee (Decision No: 2024-412, dated 10.09.2024) and complied with the Declaration of Helsinki.

## 3. Results

Among the 93 BC samples obtained from 86 patients, 74 (79.57%, n = 74/93) yielded monomicrobial and 19 (20.43%, n = 19/93) polymicrobial growth. MALDI-TOF MS identified 117 isolates comprising 82 (70.09%, n = 82/117) gram-negative, 31 (26.50%, n = 31/117) gram-positive bacteria, 2 fungi (1.71%, n = 2/117), and 1 anaerobe (0.85%, n = 1/117). The most frequently isolated pathogens were *Klebsiella pneumoniae* (25.00%, n = 29/117), *Escherichia coli* (17.24%, n = 20/117), *Staphylococcus spp.* (15.52%, n = 18/117), and *Pseudomonas aeruginosa* (10.34%, n = 12/117) (Table 2).

**Table 2:** Blood culture and BCID2 results and comparison for identification.

Empirical treatment											Norwegian Ministry of Health^[13]^	United Kingdom (Stanford University)^[14]^	
												Community	Healthcare
											Benzilpenisilin + Gentamisin	Vancomycin + (Ceftriaxone or Ertapenem or Piperacillin/tazobactam)	Vancomycin or (Piperacillin/Tazobactam ot Cefepime ot Meropenem)
	TP (n) (BC+/BCID2+)	FP (n) (BC−/BCID2+)	TN (n) (BC−/BCID2−)	FN (BC+/BCID2−)	Se (%)	Sp (%)	PPV (%)	NPV (%)	Concordance rate (%)	Concordance number (n)			
Organisms													
*Enterobacterales*	58	5	52	2	96,67%	91,23%	92,06%	96,30%	94,02%	110			
*Enterobacter cloacae kompleksi*	1	3	113	0	100,00%	97,41%	25,00%	100,00%	97,44%	114	Meropenem	Cefepime or Ertapenem or Ciprofloxacin or Levofloxacin or TMP-SMX	
*Escherichia coli*	19	0	97	1	95,00%	100,00%	100,00%	98,98%	99,15%	116		Ceftriaxone or Ciprofloxacin or Levofloxacin or Cefazolin	
*Klebsiella aerogenes*	0	0	117	0	–	100,00%	–	100,00%	100,00%	117		Cefepime or Ertapenem or Ciprofloxacin or Levofloxacin or TMP-SMX	
*Klebsiella oxytoca*	1	2	113	1	50,00%	98,26%	33,33%	99,12%	97,44%	114	Meropenem	Ceftriaxone or Ciprofloxacin or Levofloxacin or Cefazolin	
*Klebsiella pneumoniae grup*	30	0	87	0	100,00%	100,00%	100,00%	100,00%	100,00%	117		Ceftriaxone or Ciprofloxacin or Levofloxacin or Cefazolin	
*Proteus spp.*	1	0	115	1	50,00%	100,00%	100,00%	99,14%	99,15%	116		Ceftriaxone or Ciprofloxacin or Levofloxacin or Cefazolin	
*Salmonella spp.*	0	0	117	0	–	100,00%	–	100,00%	100,00%	117			
*Serratia marcescens*	1	0	116	0	100,00%	100,00%	100,00%	100,00%	100,00%	117		Cefepime or Ertapenem or Ciprofloxacin or Levofloxacin or TMP-SMX	
*Acinetobacter calcoaceticus-baumannii kompleks*	7	2	108	0	100,00%	98,18%	77,78%	100,00%	98,29%	115	Meropenem	Ask for a consultation	
*Pseudomonas aeruginosa*	11	1	104	1	91,67%	99,05%	91,67%	99,05%	98,29%	115	Meropenem	Ask for a consultation	
*Bacteroides fragilis*	0	0	117	0	–	100,00%	–	100,00%	100,00%	117	Metranidazol		
*Haemophilus influenzae*	0	0	117	0	–	100,00%	–	100,00%	100,00%	117	Sefotaksim		
*Neisseria meningitidis*	0	0	117	0	–	100,00%	–	100,00%	100,00%	117			
*Stenotrophomonas maltophilia*	3	0	114	0	100,00%	100,00%	100,00%	100,00%	100,00%	117	TMP-SMX + seftazidim-avibaktam + aztreonam	Ask for a consultation	
*Enterococcus faecalis*	3	2	111	1	75,00%	98,23%	60,00%	99,11%	97,44%	114	Ampisilin		
*Enterococcus faecium*	2	0	115	0	100,00%	100,00%	100,00%	100,00%	100,00%	117	Vankomisin		
*Listeria monocytogenes*	0	0	117	0	–	100,00%	–	100,00%	100,00%	117			
*Staphylococcus spp.*	16	2	99	0	100,00%	98,02%	88,89%	100,00%	98,29%	115			
*Staphylococcus aureus*	1	0	116	0	100,00%	100,00%	100,00%	100,00%	100,00%	117	Kloxacillin		
*Staphylococcus epidermidis*	8	1	108	0	100,00%	99,08%	88,89%	100,00%	99,15%	116			
*Staphylococcus lugdunensis*	1	0	116	0	100,00%	100,00%	100,00%	100,00%	100,00%	117			
*Streptococcus spp.*	2	0	115	0	100,00%	100,00%	100,00%	100,00%	100,00%	117			
*Streptococcus agalactiae*	0	0	117	0	–	100,00%	–	100,00%	100,00%	117			
*Streptococcus pneumoniae*	0	0	117	0	–	100,00%	–	100,00%	100,00%	117	Penisilin		
*Streptococcus pyogenes*	0	0	117	0	–	100,00%	–	100,00%	100,00%	117			
*Candida albicans*	0	1	116	0	–	99,15%	0,00%	100,00%	99,15%	116		Fluconazole or Voriconazole or Caspofungin orL-AmB	
*Candida auris*	1	1	115	0	100,00%	99,14%	50,00%	100,00%	99,15%	116		Kaspofungin	
*Candida glabrata*	0	0	117	0	–	100,00%	–	100,00%	100,00%	117		Kaspofungin or L-AmB	
*Candida krusei*	0	0	117	0	–	100,00%	–	100,00%	100,00%	117		Varikonazol or Kaspofungin	
*Candida parapsilosis*	0	0	117	0	–	100,00%	–	100,00%	100,00%	117		Fluconazole or Voriconazole or Caspofungin or L-AmB	
*Candida tropicalis*	0	0	117	0	–	100,00%	–	100,00%	100,00%	117		Fluconazole or Voriconazole or Caspofungin or L-AmB	
*Cryptococcus neoformans*/*gattii*	0	0	117	0	–	100,00%	–	100,00%	100,00%	117			
Other gram-negative bacteria not included in the BCID2 panel.*	0	0	116	1	0,00%	100,00%	–	99,15%	99,15%	116			
Other gram-positive bacteria not included in the BCID2 panel.**	0	0	112	5	0,00%	100,00%	–	95,73%	95,73%	112			
Other yeasts not included in the BCID2 panel***	0	0	116	1	0,00%	100,00%	–	99,15%	99,15%	116			
Other anaerobic bacteria not included in the BCID2 panel****	0	0	116	1	0,00%	100,00%	–	99,15%	99,15%	116			

BC = blood culture, BCID2 = BioFire FilmArray Blood Culture Identification 2, FN = false negative, FP = false positive, NPV = negative predictive value, PPV = positive predictive value, Sn = sensitivity, Sp = specificity, TN = true negative, TP = true positive.

* *Pseudomonas oryzihabitans*.

** *Leuconostoc mesenteroides*, *Bacillus cereus*, *Bacillus licheniformis*, *C. Striatum*, *Micrococcus luteus*.

*** *Candida lusitante*.

**** *Bacteroides thetaiotaomicron*.

BCID2 failed to detect any pathogen in 9 samples (9.68%, n = 9/93), but most frequently identified *K. pneumoniae* (25.64%, n = 30/117), *E. coli* (16.24%, n = 19/117), *Staphylococcus spp.* (15.38%, n = 18/117), and *P. aeruginosa* (10.26%, n = 12/117), with fungal detection in 3 samples (2.56%, n = 3/117); anaerobes were not detected. At the isolate level, BCID2 demonstrated 100% Sn and Sp for *K. pneumoniae*, *S. marcescens*, *A. baumannii*, and *Proteus spp.*; 99.18% for *E. coli*; and 97.54% for *K. oxytoca*. Agreement for Enterobacterales was 98.08% (n = 51/52). A total of 15 clinical and 8 analytical FNs were observed at this level.

**Figure 1.**
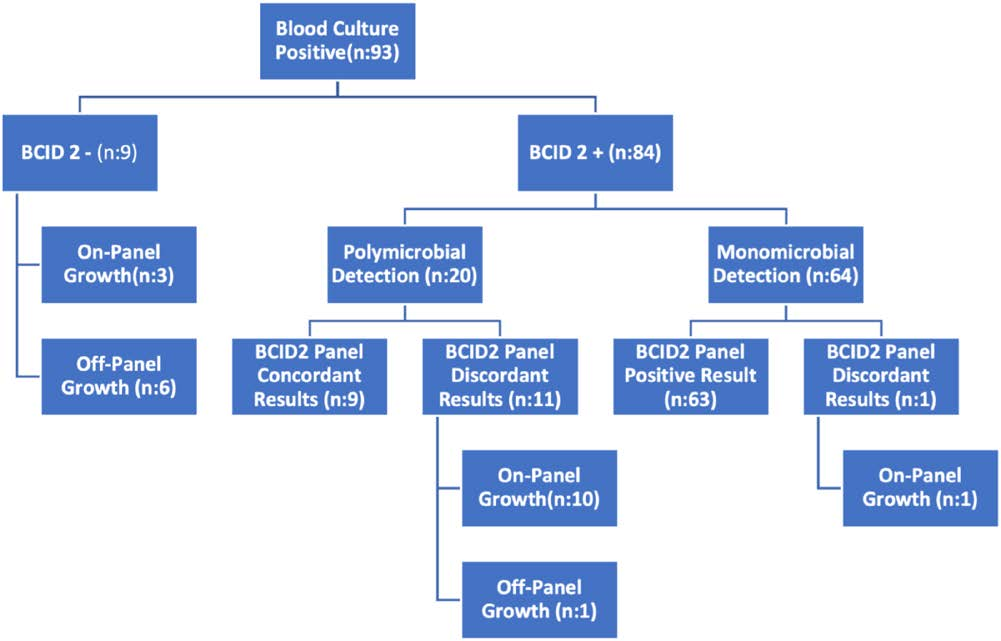
Analysis of BCID2 to blood culture results for sample-level comparison. BCID2 = BioFire FilmArray Blood Culture Identification 2.

**Figure 2.**
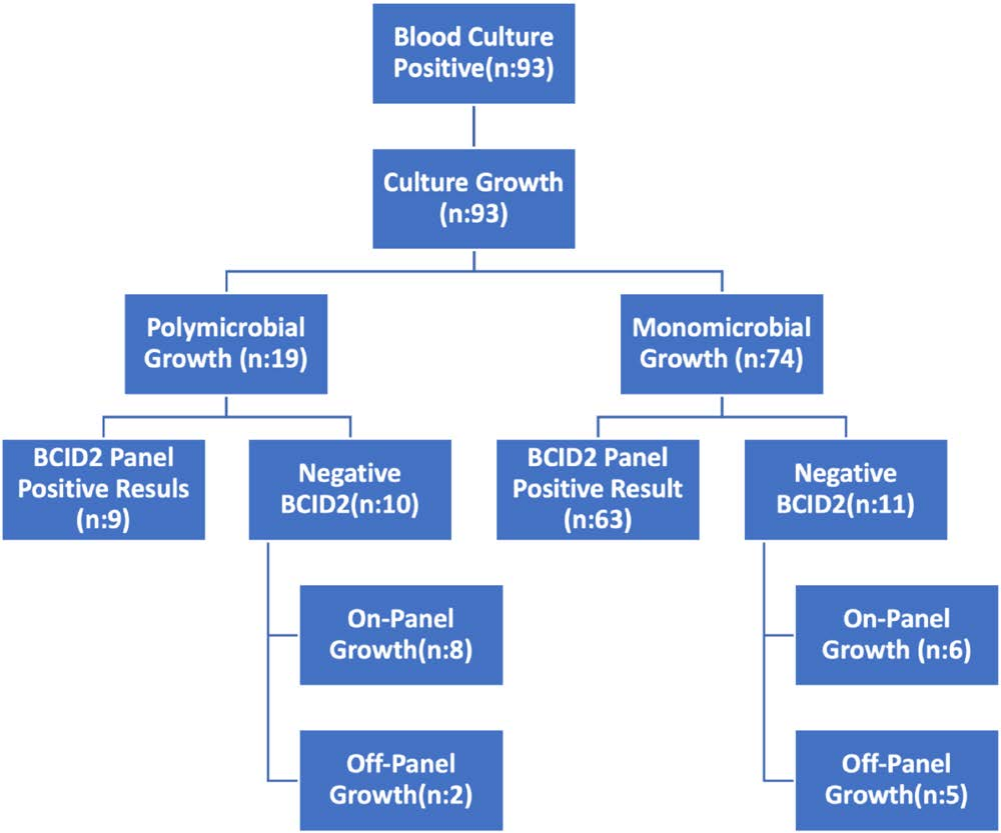
Analysis of blood culture to BCID2 results for sample-level comparison. BCID2 = BioFire FilmArray Blood Culture Identification 2.

At the sample level, analytical Sn was 83.72% (n = 72/86) and clinical Sn 77.41% (n = 72/93). BCID2 successfully identified 84 of 93 culture-positive samples (90.32%, n = 84/93). Of 20 BCID2-polymicrobial samples, 9 (45.00%, n = 9/20) were culture-confirmed, whereas 63 of 64 monomicrobial BCID2 results (98.42%, n = 63/64) matched culture (Figs. 1 and 2). Discrepancies occurred in 21 samples (22.58%, n = 21/93), 14 involving in-panel organisms and 7 involving non-panel organisms (Tables 3 and 4). These were significantly more frequent in polymicrobial samples (52.63%, n = 10/19) than in monomicrobial ones (14.86%, n = 11/74) (*P* < .05).

**Table 3:** Sample-level analysis of BCID2 result discrepancies for microorganisms included in the panel.

Isolate number	BCID2 result	Bottle type	Culture type	Gram staining	MALDI-TOF result
10	*Acinetobacter calcoaceticus-baumannii kompleks*	Aerop	Monomicrobial	GNR	*Acinetobacter baumannii* *
	*Candida albicans*				
27	*K. pneumoniae grup*	Aerop	Polymicrobial	GNR	*K. pneumoniae* *
	*Pseudomonas aeruginosa*				*Pseudomonas aeruginosa* *
	*Acinetobacter calcoaceticus-baumannii kompleks*				
32	*K. pneumoniae grup*	Aerop	Polymicrobial	GNR	*K. pneumoniae* *
	*Serratia marcesnes*				*Serratia marcesnes* *
	*E. fecalis*				
	*Acinetobacter calcoaceticus-baumannii kompleks*				
34	*K. pneumoniae grup*	Aerop	Monomicrobial	GNR	*K. pneumoniae* *
	*E. fecalis*				
37	*K. pneumoniae grup*	Aerop	Monomicrobial	GNR	*K. pneumoniae* *
	*Candida auris*				
45	*K. oxycota*	Aerop	Polymicrobial	GNR, GPC	*K. oxycota* *
	*K. pneumoniae grup*				*K. pnuomniae* *
					*S. epidermidis*
76	*K. pneumoniae grup*	Aerop	Polymicrobial	GNR, GPC	*Proteus spp*
	*E. faecalis*				*K. pneumoniae* *
					*E. faecalis* *
20	*K. pneumoniae grup*	Anaerop	Monomicrobial	GNR	*K. pneumoniae* *
	*Staphylococcus spp.*				
35	*Proteus spp.*	Aerobic	Monomicrobial	GNR	*P. mirabilis* *
	*Staphylococcus spp.*				
68	*Staphylococcus spp.*	Anaerop	Polymicrobial	GNR	S. hominis**
					S. capitis**
					*E. coli*
78	*Escherichia coli*	Aerobic	Polymicrobial	GNR	*E. coli* *
	*K. pneumoniae grup*				*K. pneumoniae* *
	*Klebsiella oxycota*				
	*Staphylococcus spp.*				
83	Negative	Pediatrik	Monomicrobial	GNR	*Pseudomonas aeruginosa*
86	Negative	Aerop	Polymicrobial	GNR, GPC	*K. oxytoca*
					*S. hemalitucus*
41	Negative	Aerop	Polymicrobial	GPC	*E. fecalis*
					*S. pettenkofori*

BCID2 = BioFire FilmArray Blood Culture Identification 2, GNR = gram-negative rod, GPC = gram-positive cocci.

* Detected by BCID2.** Detected by BCID2 at the species level.

**Table 4:** Sample-level analysis of BCID2 result discrepancies for microorganisms not included in the panel.

Sample number	BCID2 result	Bottle type	Culture type	Gram staining	MALDI-TOF result
26	Negative	Aerop	Polymicrobial	GNR	*Bacteroides thetaiotaomicron*
					*Candida lusitante*
30	*S. lugduninensis*	Aerop	Polymicrobial	GPC	*S. lugduninensis* *
	*S. epidermis*				*Leuconostoc mesenteroides*
39	Negative	Aerop	Monomicrobial	GNR	*Bacillus cereus*
42	Negative	Aerop	Monomicrobial	GNR	*Bacillus licheniformis*
44	Negative	Aerop	Monomicrobial	GPR	*C. striatum*
80	Negative	Pediatrik	Monomicrobial	GPC	*Micrococcus luteus*
90	Negative	Aerop	Monomicrobial	GNR	*Pseudomonas oryzihabitans*

BCID2 = BioFire FilmArray Blood Culture Identification 2, GNR = gram-negative rod, GPC = gram-positive cocci, GPR = gram-positive rod.

* Detected by BCID2 at the species level.

Among discrepancies due to non-panel organisms, 62.5% (n = 5/8) were gram-positive, 12.5% (n = 1/8) gram-negative, 12.5% (n = 1/8) yeast, and 11.5% (n = 1/8) anaerobe. Phenotypic resistance was identified in 63 isolates from 55 samples. At the sample level, BCID2 detected 55 resistance genes in 45 samples (48.39%, n = 45/93), with genotypic–phenotypic concordance of 73.46% (Table 5).

**Table 5:** Blood culture and BCID2 results and comparison for antimicrobial resistance.

Empirical treatment											Norwegian Ministry of Health.^[13]^	United Kingdom (Stanford University).^[14]^	
												Comminity	Healthcare
											Benzilpenisilin + Gentamisin	Vancomycin + (Ceftriaxone or Ertapenem or Piperacillin/tazobactam)	Vancomycin or (Piperacillin/Tazobactam or Cefepime or Meropenem)
	TP [ESBL/CTX-M (+)]	FP [non-ESBL/CTX-M (+)]	FN [ESBL/CTX-M (−)]	TN [non-ESBL/CTX-M (−)]	Sn (%)	Sp (%)	PPV (%)	NPV (%)	Concordance rate (%)	Concordance number (n)			
*K. pneumoniae*	18	0	4	3	81,82%	100,00%	100,00%	42,86%	84,00%	21	Meropenem		
*E. coli*	10	0	1	6	90,91%	100,00%	100,00%	85,71%	94,12%	16	Meropenem		
*K. oxycota*	0	0	0	1	–	100,00%	–	100,00%	100,00%	1	Meropenem		
*Proteus spp.*	0	0	0	1	–	100,00%	–	100,00%	100,00%	1	Meropenem		
*Enterobacter cloacae kompleksi*	0	0	0	2	–	100,00%	–	100,00%	100,00%	2	Meropenem		
*Pseudomonas aeruginosa*	0	0	0	1	–	100,00%	–	100,00%	100,00%	1	Meropenem		
	True positive [CRE/Carbapenamase genes (+)]	False positive [non-CRE/Carbapenamase genes (+)]	False negative [CRE/Carbapenamase genes (−)]	True negative [non-CRE/Carbapenamase genes (−)]	Se (%)	Sp (%)	PPV (%)	NPV (%)	Concordance rate (%)	Concordance number (n)	Meropenem		
*K. pneumoniae*	18	0	2	5	90,00%	100,00%	100,00%	71,43%	92,00%	23	Seftazidim avibaktam + Aztroenam or colistin + tigesiklin		
*E. coli*	1	1	0	15	100,00%	93,75%	50,00%	100,00%	94,12%	16	Seftazidim avibaktam + Aztroenam or colistin + tigesiklin		
*K. oxycota*	0	0	0	1	–	100,00%	–	100,00%	100,00%	1	Seftazidim avibaktam + Aztroenam or colistin + tigesiklin		
*Proteus spp.*	0	0	0	1	–	100,00%	–	100,00%	100,00%	1	Seftazidim avibaktam + Aztroenam or colistin + tigesiklin		
*Enterobacter cloacae kompleksi*	0	0	0	2	–	100,00%	–	100,00%	100,00%	2	Seftazidim avibaktam + Aztroenam or colistin + tigesiklin		
*Pseudomonas aeruginosa*	2	0	0	1	100,00%	100,00%	100,00%	100,00%	100,00%	3	(Sefiderokol or seftolozan-tazobaktam) + colistin		
*Acinetobacter baumanii*	0	0	5	0	0,00%	–	–	0,00%	0,00%	0	Colistin + tigesiklin		
	True positive [MRSA/*mecA*/*C* (−)]	False positive [non-MRSA/*mecA*/*C*}	False negative [MRSA/*mecA*/*C* (−)]	True negative [non-MRSA/Z mecA/*C* (−)]	Se (%)	Sp (%)	PPV (%)	NPV (%)	Concordance rate (%)	Concordance number (n)			
*S. epidermis*	5	1	0	0	100,00%	0,00%	83,33%	–	83,33%	5	Vankomisin	Vankomisin	Vankomisin
*S. aureus*	1	0	0	0	100,00%	–	100,00%	–	100,00%	1	Vankomisin	Vankomisin	Vankomisin
*S. hominis*	0	0	1	1	0,00%	100,00%	–	50,00%	50,00%	1			
					–	–	–	–	–	0			
					–	–	–	–	–	0			
	True positive [VRE/*vanA*/*B* (−)]	False positive [non-VRE/*vanA*/*B*}	False negative [VRE/*vanA*/*B* (−)]	True negative [non-VRE/*vanA*/*B* (−)]	Se (%)	Sp (%)	PPV (%)	NPV (%)	Concordance rate (%)	Concordance number (n)			
*E. faecalis*	0	0	0	2	–	100,00%	–	100,00%	100,00%	2	Linezolid or Daptomisin		
*E. faecium*	0	0	0	1	–	100,00%	–	100,00%	100,00%	1	Daptomisin + Ampsilin		

BCID2 = BioFire FilmArray Blood Culture Identification 2, FN = false negative, FP = false positive, NPV = negative predictive value, PPV = positive predictive value, Sn = sensitivity, Sp = specificity, TN = true negative, TP = true positive.

At the isolate level, concordance for ESBL and carbapenemase genes was 81.82% in *K. pneumoniae* and 90% for carbapenemases; 90.91% and 100% in *E. coli*; and 100% for both in *P. aeruginosa*. No resistance genes were detected in 5 *A. baumannii* isolates despite phenotypic resistance. mecA/C was detected in 5 of 6 *S. epidermidis* and in 1 *S. aureus*. Overall genotype–phenotype concordance was 88.24% (n = 68/77) (Table 6). Among 70 samples tested for both identification and resistance, 55 (36 resistant, 19 susceptible) were concordant, and 15 were discordant (13 culture-resistant, BCID2-susceptible; 2 culture-susceptible, BCID2-resistant).

**Table 6:** Comparison of BCID2 and CMs in antimicrobial resistance detection: genotypic and phenotypic concordance.

Organisms	Antimicrobial resistance genes identified by the BCID2 panel	Antimicrobial resistance phenotype identified by the conventional methods	Concordance rate (n (%))	Concordance rate (n (%))
* K. pneumoniae*	*bla*_NDM_ + *bla*_OXA48-like_ (n:8)	Carbapenem resistant, (n = 20)	100,00%	18
	*bla*_KPC_ (n:3)			
	*bla*_OXA48-like_ (n:3)			
	*bla*_KPC_ + *bla*_OXA48-like_ (n:1)			
	*bla*_VIM_ + *bla*_NDM_ + *bla*_OXA48-like_(n:1)			
	*bla*_NDM_ (n:1)			
	*bla*_CTX-M_ (n:18)	ESBL (n:22)	100,00%	18
* E. coli*	*bla*_NDM_ (n:1)	Carbapenem resistant, (n = 1)	50,00%	1
	*bla*_OXA48-like_ (n:1)			
	*bla*_CTX-M_ (n:10)	ESBL (n:11)	100,00%	10
* P. aeruginosa*	*bla*_NDM_ (n:2)	Carbapenem resistant, (n = 2)	100,00%	2
* A. baumanii*	*bla*_IMP_, *bla*_KPC_, *bla*_OXA48-like_, *bla*_NDM_, *bla*_VIM_: 0	Carbapenem resistant, (n = 5)		5
* S. epidermis*	*mecA*/*C* (n:5)	Methicillin resistant (n = 5)	100,00%	5
* S. aureus*	*mecA*/*C* and *MREJ* (n:1)	Methicillin resistant (n = 1)	100,00%	1
* Staph spp. (S. hominis*)	*mecA*/*C* and *MREJ* (n:0)	Methicillin resistant (n = 1)		0
General concordance			88,24%	68

BCID2 = BioFire FilmArray Blood Culture Identification 2.

**Figure 3.**
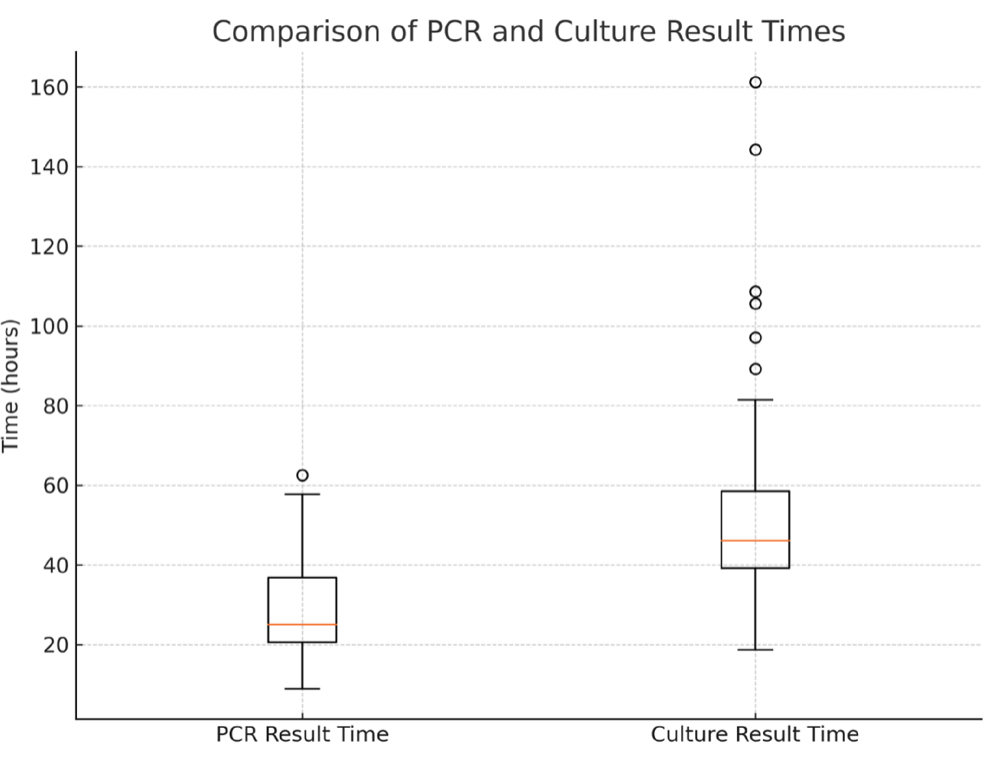
Comparison of BCID2 and culture result times. BCID2 = BioFire FilmArray Blood Culture Identification 2.

BCID2 results were reported in a mean of 1 day 4 hours 52 minutes (SD: 12h10min; range: 8 h 55 min–2 d 14 h 31 min), significantly faster than CTMs (mean: 2 d 4 h 9 min; SD: 22 h 55 min; range: 18 h 38 min–6 d 17 h 9 min), with a mean difference of 23 hours 17 minutes (*P* < .001) (Fig. 3).

Sample-level agreement by bottle type was 90.00% (n = 18/20) for anaerobic, 77.77% (n = 7/9) for pediatric, and 73.43% (n = 47/64) for aerobic bottles (*P* > .05). Agreement by gram stain was 100% for gram-positive coccobacilli (n = 1/1), 94.1% for gram-positive cocci (GPC) (n = 16/17), 79.1% for gram-negative bacilli (n = 53/67), and 0% for gram-negative cocci (n = 0/2) (*P* < .001). Empirical treatment simulations based on the Norwegian national guideline^[13]^ and the UK (Stanford) guideline^[14]^ showed that appropriate therapy could be selected in 85.00% of patients (n = 79/93) using BCID2 species identification alone. When resistance gene data were included, appropriate treatment based on the Norwegian guideline increased to 91.5% (n = 107/117) at the isolate level. The UK guideline, which applies only to MRSA, allowed correct therapy in 77.7% of cases (n = 7/9). When both identification and resistance data were used, BCID2 enabled appropriate treatment modification in 70.85% of patients (n = 60/85) at the sample level. According to the Norwegian guideline, empirical therapy alone would have failed in 29.15% of cases (n = 25/85).

## 4. Discussion

Bacteremia is a significant cause of mortality, with global rates ranging from 10 to 28%.^[18–20]^ Although early administration of appropriate antimicrobial therapy improves survival, empirical treatment remains appropriate in only 60 to 70% of cases.^[19]^ Thus, timely microbiological diagnosis is crucial for improving outcomes. IDSA guidelines recommend rapid diagnostic tests to optimize antibiotic therapy.^[21]^ Supporting data show that BCID2-guided management shortens time to appropriate treatment and reduces mortality in critically ill bacteremia patients.^[22]^

This study compared the BioFire FilmArray BCID2 panel with CTMs and assessed its impact on sepsis treatment using guideline-based simulation. Ninety-three samples were analyzed with both methods. Both identified non-fermentative gram-negative bacteria, *Enterobacterales*, and *Staphylococcus spp*., and detected carbapenem, beta-lactam, and methicillin resistance. Vancomycin resistance was undetectable, and colistin resistance could not be reciprocally evaluated. Previous studies reported BCID2 Sn at the isolate level over 90% (50–100%), consistent with the present findings.,^[16,23,24]^

Agreement rates between BCID2 and QC methods vary. A Turkish study reported 95.3% agreement in monomicrobial and 79% in polymicrobial samples. Berinson et al found 88.3% in monomicrobial and 61.3% in polymicrobial samples; Sparks et al reported 92.9% and 28.6%, respectively.^[4,25,26]^ In this study, BCID2 showed 85.13% agreement in monomicrobial and 47.36% in polymicrobial samples, indicating decreased performance in complex infections. Although BCID2 performs well diagnostically, reduced Sn in polymicrobial cases warrants cautious interpretation and highlights the need for panel improvements or complementary diagnostics.

FNs in polymicrobial samples may stem from low microorganism concentrations, undetectable by BCID2 or gram stain.^[4]^ At the isolate level, study findings align with manufacturer-reported sensitivities.^[27]^ Analytical Sn was 83.72% (72/86 samples); 14 samples (8 polymicrobial, 6 monomicrobial) yielded FNs despite culturable organisms. Additionally, BCID2 identified ten microorganisms in 8 samples undetected by culture – six with 1 false-positive target, 2 with 2. Eight culture-isolated organisms undetected by BCID2 caused FNs in 5 samples. These findings, rare in literature, underscore the need to assess BCID2-detected FPs. In these 8 samples, pathogens were absent on gram stain, suggesting BCID2 may detect low-concentration organisms missed by CTMs, though confirmation requires further studies.

Incompatibility frequency may vary by isolate. A prior study cited E. coli as frequently associated with sample-level incompatibility and isolate-level FNs, attributed to operator error or PCR Sn variation, not rare genotypes.^[16,24]^ In one study, FNs were confirmed by re-culturing and rerunning BCID2, which successfully detected all strains.^[24]^ In the current study, *E. coli* (n = 1), *Proteus spp.* (n = 1), *K. oxytoca* (n = 1), and *P. aeruginosa* (n = 1) caused FNs, while the most frequent FPs were from *Enterobacter cloacae* complex (n = 3).

Studies have noted limitations in BCID2 clinical Sn due to its panel scope. Some suggest adding anaerobes and rare gram-negatives to enhance accuracy.^[16]^ These limitations may be mitigated when BCID2 is used alongside culture methods.^[24]^ In this study, incompatibilities involved *Candida lusitaniae*, *Bacteroides thetaiotaomicron*, and *Pseudomonas oryzihabitans* genera partially represented in BCID2. Expanding panel content is recommended. Earlier studies proposed that genus-level *Bacteroides* and anaerobes like *Clostridium spp*. be added, though others found current limitations acceptable when combined with CTMs.^[16,24]^ Our findings stress the importance of extending molecular panels for key anaerobes and gram-negatives. BCID2’s fixed structure limits detection of rare species, warranting future research to balance diagnostic scope with clinical relevance.

BCID2 Sp has ranged from 96 to 100% in prior studies.^[24,28]^ Our results (analytical Sp 99.47%, clinical Sp 99.52%) align with these, confirming BCID2’s robustness against contamination and technical artifacts. This high Sp affirms BCID2’s reliability, but clinical context must guide interpretation to avoid misdiagnosis in complex cases.

Identifying contamination in BCs is crucial to avoid unnecessary treatments and diagnostics.^[29]^ Multiple BC sets and integration of lab and clinical data enhance assessment accuracy. In this study, 9 samples were deemed contaminants, with no further action. Misidentification risks inappropriate therapy and extended hospitalization. Although BCID2 is reliable, corroboration with serial BCs and clinical data remains essential, as distinguishing contaminants remains difficult in molecular systems.

Concordance between BCID2-detected resistance genes and phenotypic resistance was 87.04% in enteric and 85.71% in gram-positive bacteria. A local study reported 100% concordance in both. However, in *Acinetobacter baumannii*, resistance gene detection by BCID2 was 0%, consistent with local literature.^[4]^ While BCID2 identified *A. baumannii* species with 100% Sn (also reported by Salimnia, Rule, and Fhooblall as 100%, 90%, and 66.7%, respectively),^[30–32]^ it failed to detect resistance genes. This is attributed to the diversity of resistance mechanisms in *A. baumannii*, which are not fully covered by the BCID2 panel. Mechanisms include β-lactamases, aminoglycoside-modifying enzymes, efflux pumps, and porin loss.^[33]^ BCID2 detects genes like *bla*_IMP_, *bla*_KPC_, *bla*_OXA48-like_, *bla*_NDM_, *bla*_VIM_, *mcr-1*, and *bla*_CTX-M_. This underscores that molecular tests may not fully reflect resistance profiles in multi-mechanism bacteria. Hence, BCID2 must be supplemented by phenotypic methods in carbapenem-resistant strains. Future improvements should expand resistance gene panels for better diagnostic and therapeutic accuracy.

Timely pathogen identification is vital in sepsis.^[34]^ Rapid detection of BSIs improves antibiotic use and outcomes.^[3]^ Studies show BCID2 significantly reduces time to result versus culture. A Turkish study reported BCID2 was 1 day, 5 hours, and 35 minutes faster^[4]^; an international study reported a 2-day, 3-hour, and 17-minute difference.^[30]^ Sparks et al found BCID2 gave results in 24.6 ± 16.8 hours vs 38.2 ± 21.9 hours for culture.^[35]^ Another study reported 21 versus 49 hours, respectively.^[36]^ In this study, BCID2 was 23 hours and 17 minutes faster than CTMs (*P* < .001). Limitations included BCID2’s single-system design, limiting parallel processing. Despite this, BCID2’s time advantage reinforces its utility in accelerating diagnosis and treatment decisions.

It is noteworthy that, while the overall turnaround time was consistently reduced with BCID2, the distribution of result times also differed substantially between methods. Several extreme values exceeding 100 hours were observed in the culture group, likely reflecting delays due to subculturing, slow-growing or fastidious organisms, extended incubation requirements (especially for anaerobes), or repeat testing necessitated by contamination or inconclusive gram stain results – factors inherent to the conventional workflow. Conversely, the BCID2 group demonstrated a reduced number of outliers that were less pronounced, a phenomenon that may be ascribed to logistical factors such as batching schedules or sporadic instrument downtime, as opposed to diagnostic constraints. The narrower interquartile range and lower median in the BCID2 cohort further underscore its clinical advantage in delivering more rapid and consistent diagnostic outcomes.

The integration of BCID2 into routine diagnostic processes is a notable application with the potential to enhance antibiotic stewardship, reduce inappropriate antibiotic use, and improve patient outcomes. A Norwegian study reported that appropriate antimicrobial treatments arranged according to national guidelines provide better clinical outcomes compared to treatments without guidelines.^[37]^ Furthermore, it has been documented that the limitation of empirical treatments has been shown to decelerate the development of AMR.^[38]^ A further study undertaken in Norway revealed that 27% (n = 40) of recommended empirical treatments were inadequate.^[24]^ This situation reveals that recommended empirical treatments and the antibiotics to be selected after resistance and species determination may differ between countries. The variations in empirical treatment suitability reported in different studies underscore the necessity to monitor region-specific resistance data and to harmonize guidelines with local data. The in vitro BCID2 results obtained in our study clearly demonstrated the potential of this molecular test to contribute to clinical treatment decisions. When bacterial genus and species determination is taken into account, treatment of 85% (n = 79) of patients can be arranged correctly based on the United Kingdom (Stanford University) and Norwegian guidelines; thus, optimization or eradication can be achieved approximately 1 day earlier compared to CTMs based only on bacterial type.^[13,14]^ When evaluated in terms of resistance status, the United Kingdom (Stanford University) guideline only offers recommendations for the presence of MRSA, and 77.7% (7 cases) of the 9 cases in which MRSA was detected in our study were treated correctly according to BCID2 results. In contrast, the Norwegian guideline encompasses recommendations for all resistance statuses identified by the BCID2 panel. When this guideline is taken as the basis, a treatment rate of 91.5% (107 of 117 cases) was achieved in the present study. When resistance and species determination were evaluated in tandem, appropriate treatment could be determined for 70.85% (n = 60/85) of the samples in accordance with BCID2 results, as outlined in the Norwegian guideline. When evaluated in terms of empirical treatment suitability, this rate was predicted to be insufficient at 29.15% in the present study; this rate is largely parallel to the Norwegian data. These findings indicate that rapid molecular diagnostic tests such as BCID2 can make significant contributions to the optimization of antimicrobial therapy when used in conjunction with local or national antibiotic guidelines. It is imperative to acknowledge that dominant species and resistance profiles exhibit significant variations between countries and health centers. Consequently, the development of data-based guidelines tailored to the specific needs of each country and health center is of paramount importance. The expansion of the resistance gene coverage of BCID2, particularly for bacteria exhibiting intricate resistance mechanisms such as *A. baumannii*, is poised to enhance diagnostic precision and the efficacy of treatment guidance. In conclusion, the integrated use of rapid diagnostic tests with locally adapted antimicrobial stewardship programs has the potential to improve patient outcomes and slow down the development of AMR.

Limited data exist on how BC bottle type and gram stain pattern affect BCID2-CTM agreement. This study pioneered in evaluating these variables. Agreement with CTM was 90% (n = 18) in anaerobic, 77.77% (n = 7) in pediatric, and 73.43% (n = 47) in aerobic bottles; the difference was not significant (*P* > .05). However, agreement varied significantly by gram stain (*P* < .001), highest for GPC (94.1%) and gram-positive coccobacilli (100%). Thus, bottle type has no significant effect, but gram stain pattern does. Low agreement in gram-negative cocci and mixed samples (gram-negative bacilli + GPC) suggests limitations in polymicrobial or atypical morphology detection. Conversely, high GPC agreement supports BCID2’s strength for gram-positive pathogens.

This retrospective study had limitations. Samples with discordant BCID2 and culture results could not be re-analyzed. Also, the lack of a national guideline hindered integration into clinical decisions. Lastly, the absence of real-time treatment data prevented evaluation of treatment effectiveness and timing on patient outcomes.

## 5. Conclusion

In conclusion, the BCID2 system has been demonstrated to significantly accelerate the process of pathogen detection, reinforce infection control strategies, and enable timely implementation of adjustments to antimicrobial treatment. However, the system’s inability to detect certain resistance genes and polymicrobial agents highlights the necessity of employing molecular-based diagnostic methods in conjunction with conventional microbiological techniques to ensure comprehensive diagnostic accuracy. It is anticipated that future targeted expansions in the panel content and integration with real-time clinical decision support systems will further strengthen the clinical contribution of BCID2. This approach will pave the way for more effective and sustainable use of molecular diagnostic technologies, both in terms of reaching accurate and rapid diagnoses and implementing effective and rational treatment strategies.

## Author contributions

**Data curation:** İIsmail Selçuk Aygar.

**Formal analysis:** İIsmail Selçuk Aygar.

**Funding acquisition:** İIsmail Selçuk Aygar.

**Investigation:** İIsmail Selçuk Aygar.

**Methodology:** İIsmail Selçuk Aygar.

**Project administration:** İIsmail Selçuk Aygar.

**Resources:** İIsmail Selçuk Aygar.

**Software:** İIsmail Selçuk Aygar.

**Supervision:** Tuğrul Hoşbul.

**Validation:** Tuğrul Hoşbul.

**Visualization:** Tuğrul Hoşbul.

**Writing – original draft:** İIsmail Selçuk Aygar, Tuğrul Hoşbul.

**Writing – review & editing:** İIsmail Selçuk Aygar, Tuğrul Hoşbul.
